# Brain activity and the production of written text during mediumistic trance: A controlled EEG study

**DOI:** 10.1371/journal.pone.0343216

**Published:** 2026-02-24

**Authors:** Kleber Monteiro Pinto, Thaise G. L. de O. Toutain, Hugo Saba, Marco Aurélio Vinhosa Bastos Jr, Raphael Silva do Rosário, Jéssica Plácido, Naíma Loureiro, Valéria C. Fernandes, José Garcia Vivas Miranda

**Affiliations:** 1 Laboratory of Biosystems, Physics Institute, Federal University of Bahia, Salvador, Brazil; 2 Department of Education, Campus I, State University of Bahia, Salvador, Brazil; 3 School of Health and Natural Sciences, Catholic University of Salvador, Bahia, Brazil; 4 Institute of Mathematical and Computer Sciences, University of São Paulo, São Paulo, Brazil; 5 Department of Exact and Earth Sciences, State University of Bahia, Salvador, Brazil; 6 Senai Cimatec University, Salvador, Bahia, Brazil; 7 School of Medicine – Federal University of Mato Grosso do Sul, Campo Grande, Brazil; 8 Bahiana School of Medicine and Public Health, Psychology Program, Bahia, Brazil; 9 Health Sciences Institute, Federal University of Bahia, Salvador, Bahia, Brazil; 10 Electrochemistry, Energy and Materials Research Group (GPEEM), Department of Physical Chemistry, Federal University of Bahia, Institute of Chemistry, Salvador, Bahia, Brazil; 11 Postgraduate Program in Energy and Environment (PGEnAm), Federal University of Bahia, Salvador, Brazil; Tel Aviv university, ISRAEL

## Abstract

Mediumistic trance, studied as a cultural phenomenon, is considered an altered state of consciousness with dissociative and non-pathological characteristics. Studies on the effect of this trance state on linguistic performance using electroencephalography (EEG) are still scarce. We investigated the brain electrical activity of mediums using quantitative EEG (qEEG) during writing in waking and trance, and compared them with a matched control group performing simulated mediumistic writing and waking writing (n = 9 per group). EEG power differences exhibited increases across theta, alpha, beta1, and beta2 bands (p < .05) distinguishing activity during trance writing from the simulated mediumistic writing conditions. For the textual evaluations, within-group analyses showed that mediums’ trance texts scored higher than their waking texts. In contrast, no significant intergroup differences in overall text quality emerged between mediums and controls. Together, these findings support the view that complex linguistic production is preserved across groups, while trance is associated with distinct spectral dynamics during writing in the mediums’ group. Further research is warranted to refine the characterization of neural patterns supporting linguistic production in altered states of consciousness.

## Introduction

Mediumistic trance refers to a complex set of experiential, behavioral, and physiological changes in which an individual – the medium – reports entering a distinctive state of consciousness in which verbal, motor, or written expressions are perceived as guided or controlled by an external source, often identified as a deceased personality or spiritual entity [1]. Historically, such experiences have been documented across diverse cultural and religious contexts, including Afro-Brazilian traditions, Kardecist Spiritism, shamanic practices, and other forms of possession and channeling reported worldwide [2–4,5]. In these settings, trance is not regarded as pathological but as a structured, socially sanctioned, and often therapeutically oriented phenomenon [1,3,5].

Phenomenologically, mediumistic trance involves marked alterations in attention, perception, and sense of agency, frequently accompanied by partial amnesia, changes in voice or handwriting, automatisms, and the subjective experience of an alternative identity that coexists with the medium’s ordinary self [6–9,10]. The medium often reports a state of divided or shared awareness, where personal control and an external influence are simultaneously perceived [7,5,11].

From the standpoint of consciousness research, mediumistic trance can be classified within the spectrum of Altered States of Consciousness (ASC), as originally proposed by Tart [8] and expanded by Revonsuo et al. [9], encompassing reversible, structured deviations from ordinary waking consciousness. Recent conceptual models, such as the Consensus Taxonomy of Altered States of Consciousness [12], situate mediumistic and possession trances as culturally mediated subtypes of non-ordinary states characterized by distinctive phenomenological and neurophysiological profiles. In this regard, some authors prefer the term “non-ordinary states of consciousness", emphasizing an expansion of the experiential repertoire rather than a qualitatively distinct alteration [13]. This view is further supported by contemporary neurophenomenological studies that highlight both the diversity and the internal coherence of trance-related neural patterns [14–16]. Empirical evidence suggests that individuals who engage in mediumistic practice frequently exhibit high levels of spirituality, social functioning, and well-being, without evidence of psychopathology or maladaptive dissociation [1,3,4,6,7,11,15]. Collectively, these data support the interpretation of mediumistic trance as an adaptive, culturally shaped expression of dissociative capacity, distinct from pathological forms and potentially linked to positive health outcomes.

Despite the growing body of work, scientific understanding of brain activity during mediumistic trance remains limited, particularly regarding physiological correlates and electroencephalographic (EEG) evidence [7,17]. Among EEG studies, several have employed quantitative EEG (qEEG) to examine differences in neural activity during trance [17–21]. Spectral comparisons frequently report increases in theta, alpha, and beta activity during trance [17–20], although effects vary according to study protocols. Methodological heterogeneity across tasks, acquisition parameters, and analytic approaches, however, has limited convergence on a canonical electrophysiological signature of mediumistic trance.

A notable gap concerns the investigation of cognitive processes during mediumistic trance using EEG, particularly those focusing on verbal language. Written textual production in trance, commonly identified by mediums as psychography, is one prominent expression of this phenomenon and is often characterized by well-structured, legible narratives. Mediums sometimes claim to be unaware of the content or grammar of these texts, which are attributed to deceased personalities or spirits [6]. For many years, researchers have sought to identify the cerebral correlates of language abilities across different contexts [22]. Since language represents one of the most complex emergent properties of the human brain and engages virtually the entire cortex [23], the neural correlates of a context such as psychography remain largely unexplored, as does the question of whether this phenomenon may influence the delicate balance of cortical activation underlying linguistic processing.

Language production and comprehension are broadly distributed across frontotemporal networks and multiple frequency bands [24]. Writing, specifically, engages lateralized activation in regions including the dorsal and ventral premotor cortex, Broca’s area, the supplementary motor area (SMA), and the posterior segments of the middle and inferior temporal gyri [25,26]. Researchers have employed qEEG to map frequency-specific neural activity associated with verbal language processing [27–29]. Evidence indicates that oscillatory activity across all frequency bands contributes to diverse cognitive operations [28]. Accordingly, EEG signals can be characterized by their frequency components within these functional bands relevant to linguistic performance [30]. Beta and gamma activity are often linked to cortical activation and network integration supporting language comprehension and production, whereas delta and theta activity can reflect broader integrative or inhibitory control depending on task demands [30,31], consistent with language as a complex and highly organized form of conscious activity [32].

In this context, qEEG investigations of linguistic activity during mediumistic trance may offer critical insights into the spectral characteristics and functional significance of brain oscillations. Notably, considering prior research employing qEEG in studies of both mediumship [17–21,33] and verbal language [34–39], we hypothesize that psychography in mediums entails alterations in neural oscillatory dynamics and, more specifically, exhibits an increase in slow-frequency brain activity during trance states associated with the production of texts attributed to deceased individuals. Accordingly, a central question guiding our study is whether this modulation can occur without measurable impairment in linguistic performance. Such a finding would suggest a distinctive cortical reconfiguration that preserves language processing efficiency.

Building on these premises, the present study aims to quantify EEG spectral characteristics during mediumistic psychography; examine whether slow-frequency activity (delta/theta) increases during trance relative to non-trance states; and assess whether linguistic performance remains preserved during psychographic text production. By integrating cultural practices with cognitive neuroscience, this work seeks to advance neurophenomenological models of ASC and contribute empirical evidence to the underexplored interface between mediumistic trance and language processing.

## Materials and methods

### Participants

The group of mediums (GM) consisted of ten experienced psychographic Spiritist Brazilian mediums (seven men and three women over the age of 18) from different Brazilian states. Recruitment was carried out through referrals and contact with representative bodies of different Spiritist institutions known for performing psychography.

The exclusion criteria included participants who used illicit drugs or other psychoactive substances, including cigarette smoking, due to potential confounding effects on EEG measurements; received payment for mediumistic activities, to reduce heterogeneity related to professionalization and expectancy/motivational factors (and consistent with Spiritist norms framing mediumship as charitable service); and mental disorders. These mental disorders were assessed through an interview with a psychologist and the application of the following psychological assessment instruments: Structured Clinical Interview for DSM-5 Disorders [40]; Positive and Negative Syndrome Scale (PANSS) [41]; and Brief Neuropsychological Assessment (NEUPSILIN) [42,43]. The psychological assessment instruments are shown in Supplementary Material (S1 in S1 File). Inclusion criteria required participants to be adults (≥18 years old) with at least five years of experience in mediumistic practice, regularly engaging in mediumistic writing (psychography), and producing a minimum of one text per month. Additionally, participants were required to demonstrate good mental health, as confirmed by the psychologist-conducted clinical interview and the standardized instruments listed above.

A control group (CG) composed of ten adults was included for comparison. Participants were recruited using the same procedure applied to mediums and were matched to GM by age, gender, and educational level, used as a criterion to ensure similar writing proficiency, as well as by the duration of involvement in spiritual activities typical of Spiritist communities. The CG met the same inclusion and exclusion criteria as the GM, except for experience with trance and psychography.

It is worth highlighting that the Human Research Ethics Committee at the Federal University of Bahia – Brazil authorized this study, as recorded in the approval opinion (CAAE: 24603119.3.0000.5531). Recruitment began on March 1, 2022, and concluded on August 31, 2022. All participants signed a free and informed consent form.

### Experimental procedure

**Fig 1:**
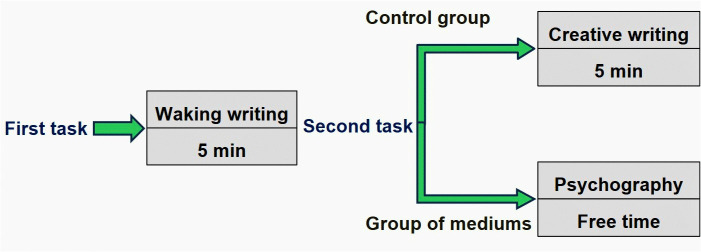
Sequence of each task with the corresponding duration for EEG recording. All tasks were performed with eyes closed. For the waking writing and creative writing tasks, participants were asked to write for a minimum of 5 min. In contrast, the psychography (PSYC) task had no time constraints, including no minimum duration, to preserve the authenticity of the trance experience.

The experimental research involving participants from both groups consisted of two stages, each with its own data collection protocol. Stage 1 consisted of conducting an introductory interview with a psychologist, followed by the application of a sociodemographic questionnaire, and different psychological and neuropsychological assessment instruments (mentioned previously). Then, Stage 2 was carried out:

1. Application of a pre-collection questionnaire to verify if the participants met the requirements for EEG collection and application of the Dissociative Experience Scale (DES) [44] (Supplementary Material, S1 and S2 in S1 File).
2. EEG data were collected during the following tasks: 1. Waking Writing (WW) and 2. Divided by group into: Creative Writing (CW), which was restricted to the control group, and Psychography (PSYC), which was restricted to the medium group (see Fig 1). Although resting-state EEG data was available, it was not analyzed in this study.

For the first written task (WW), participants from both groups were asked to write a letter to a recipient of their choice. The second written task was specific to each group. For the control group, participants were asked to perform the CW task, which consisted of writing a text as if authored by a deceased personality of their choice, simulating psychography. The medium group performed the PSYC task. Participants in this group wrote while in a mediumistic trance state, according to their usual practices for this type of production. All tasks were carried out with eyes closed throughout the writing period and by hand (pen and paper), relying on tactile guidance. This procedure was adopted both to increase consistency across participants and to reduce EEG artifacts from eye movements, as most mediums typically perform psychography with their eyes closed. Both groups performed a shared baseline (WW). Mediums then carried out psychography in trance, while controls completed a content-matched CW task (same topics/genres) without entering an altered state.

1. After completing the entire collection protocol, a questionnaire was administered to the GM to evaluate the quality of mediumistic trance experience (Supplementary material S3 in S1 File).

### Data recording and analysis

The subjects were evaluated using two approaches: brain activity using a 64-channel electroencephalogram (EEG) and textual quality analysis performed by evaluators experienced in text correction and revision.

### EEG signal processing

The parameters for EEG recording were the same for all tasks: use of a 64-channel cap (Neuvo 64-Channel Amplifier, Compumedics), following the International System (SI) 10−10 with the Cz electrode as the reference point; a sampling rate of 1,000 Hz. Electrode impedances were maintained below 50 kΩ, in accordance with the specifications of the high-input-impedance Neuvo 64-channel amplifier (Compumedics Neuroscan), which is designed to operate reliably within this range without loss of signal quality. Were excluding the reference electrode (Cz), those that did not provide measures relevant to this study (VEOG, HEOG, M1, M2, EKG, EMG, Cb1, and Cb2), and others that exhibited issues in capturing brain activity (Fz, F11, F12, FT11, and FT12), remaining 54 electrodes as following: Fp1, Fpz, Fp2, AF3, AF4, F7, F5, F3, F1, F2, F4, F6, F8, FC5, FC3, FC1, FCz, FC2, FC4, FC6, T7, C5, C3, C1, C2, C4, C6, T8, TP7, CP5, CP3, CP1, CPz, CP2, CP4, CP6, TP8, P7, P5, P3, P1, Pz, P2, P4, P6, P8, PO7, PO3, POz, PO4, PO8, O1, Oz and O2. The EEGLAB®, a MATLAB® tool, was used for data processing. Because the Brazilian mains frequency is 60 Hz – within the gamma range – and to avoid distortions from aggressive 60 Hz notch filtering, we did not analyze gamma-band activity. The data were filtered with a bandpass filter between 0.5 and 48 Hz. Next, independent component analysis (ICA) was applied using a script to automatically classify, select, and remove components that were ocular or muscular artifacts. The data were separated into 1.28-second epochs. Epochs in which the signal exceeded the threshold of ±100 μV were considered to contain artifacts and were eliminated. After these procedures, a visual inspection was performed, and any remaining epochs with artifacts were removed. Finally, to ensure homogeneity, the first 80 epochs (~ 1 min 40 s) of each record were selected for qEEG analysis.

### Text evaluation

Three Portuguese language professors who are specialists in theoretical and/or applied linguistics and have experience in text correction and revision evaluated the collected texts. For this purpose, a model to evaluate the quality of written texts was developed by the researchers. This model is grounded in linguistic frameworks [45–48] and in the criteria adopted in a similar study design on psychography employing neuroimaging [6]. These models consider the components of the language rule system. The goal is to classify writing skills and quality in a more objective and measurable way.

The model established six criteria: spelling accuracy, lexical item selection, grammatical accuracy, text organization, textual cohesion, and textual coherence. Scores ranged from 1 (insufficient) to 4 (very good) for each criterion. Considering the need to avoid bias from subjective evaluations of text quality, the criterion of assessing thematic development and complexity was not adopted. The evaluation procedure used “blinded” texts, so the professors evaluating them did not know which participant had written the text, which group they belonged to, or what type of writing task had been performed. The Text Evaluation Model can be found in Supplementary Material S4 in S1 File.

The written productions were summarized as short narratives, considering time and word count.

### Statistical analysis

A statistical analysis of the EEG data was performed based on the organization of the electrodes, which were distributed globally and separated by cerebral hemisphere: right hemisphere (RH) (Fp2, AF4, F2, F4, F6, F8, FC2, FC4, FC6, C2, C4, C6, T8, CP2, CP4, CP6, TP8, P2, P4, P6, P8, PO4, PO8 and O2) and left hemisphere (LH) (Fp1, AF3, F7, F5, F3, F1, FC5, FC3, FC1, T7, C5, C3, C1, TP7, CP5, CP3, CP1, P7, P5, P3, P1, PO7, PO3 and O1).

Considering the analysis of brain activity during written production in a mediumistic trance state, the fundamental comparisons were between groups by task and between tasks within each group. The variables analyzed in terms of power density of the frequency bands were: Delta (0.5–3.99 Hz), Theta (4–7.9 Hz), Alpha (8–13 Hz), Beta 1 (13–19 Hz), and Beta 2 (20–30 Hz).

We used a mixed ANOVA of repeated measures for comparisons. To verify the reliability of normality, joint variability of the variables, homogeneity, and sphericity of the distribution, we applied Box’s M test, Levene’s homogeneity test, and Mauchly’s sphericity test. To control for multiple comparisons, we applied Bonferroni correction to the pos-hoc pairwise comparisons of the ANOVA of mixed repeated measures. Pairwise tests (Welch-corrected t-tests or Wilcoxon, as appropriate) are reported with Bonferroni-adjusted p-values, and p values ≤ 0.05 were considered significant. All statistical analyses were performed using SPSS® software.

A mixed repeated measures ANOVA was also performed to evaluate the quality of the texts, along with Box’s M test, Levene’s test, and Mauchly’s test. This was done to compare the scores of the content written by the two groups. The sample resulting from the total writing tasks was 36 texts (CG: nine WW texts + nine CW texts; GM: nine WW texts + nine PSYC texts), with three corresponding scores assigned by each of the three evaluators. Additionally, one-factor ANOVA and a posteriori test with Bonferroni corrections were used to compare evaluators based on their scores, assessing homogeneity in evaluation distribution among evaluators.

## Results

### Behavioral data

In line with the inclusion criteria of no psychopathological markers, the psychological and neuropsychological evaluations conducted in the first stage indicated that mediums presented healthy cognitive functions, with no observed behavioral issues or indications of mental disorders (Supplementary Material S1 in S1 File). Beyond serving as a prerequisite for participation, these findings corroborate evidence from previous studies indicating a positive association between mediumistic experiences and better outcomes in mental health, physical health, social adjustment, and well-being [1–4,6,7,49].

### Text evaluation

The texts produced during the WW tasks (performed by both groups) and the CW tasks (restricted to the CG) took, on average, 5–7 minutes (EEG recording time) and contained approximately 120 words. Texts produced during the PSYC task (restricted to the GM) were generated over five to ten minutes of EEG recording and averaged 244 words in length.

**Fig 2:**
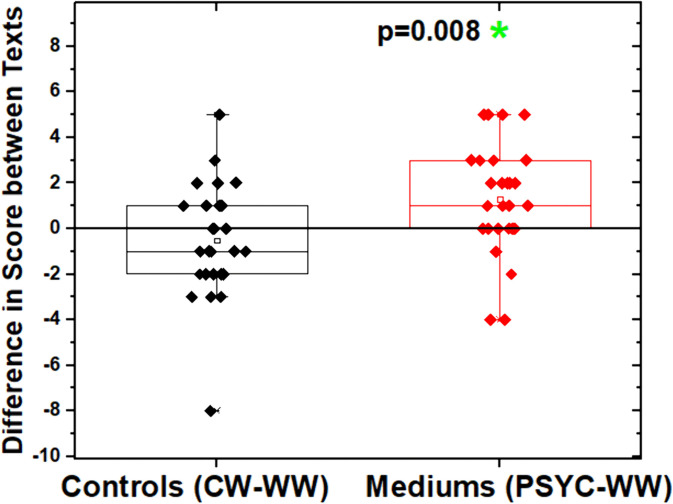
Intragroup comparisons. In the CG, no significant difference was observed between the CW and WW tasks, whereas in the GM a significant difference was found between the PSYC and WW tasks, as indicated by post hoc analysis (p = 0.008).

Assuming homogeneity of variances, post hoc tests revealed no differences among the three evaluators, indicating consistency in score distribution and supporting the reliability of the ratings. Regarding text scores, based on evaluations from the three examiners, a significant task-by-group interaction was observed (F(1, 52) = 7.449, p = 0.009). Post hoc analysis showed a difference (p = 0.008) only between the WW and PSYC tasks within the GM group (Fig 2).

Therefore, only the GM showed a significant change in text quality between waking and trance states, with higher scores for PSYC texts. This is evident in the positive difference in average score between PSYC and WW. Conversely, there was no significant difference in the comparison between the WW and CW tasks of the CG. Thus, the quality of texts produced by non-mediums remains stable, regardless of psychography simulation. Additionally, there was no significant difference in text evaluation comparisons between groups (CG *vs.* GM) involving all tasks.

Another finding was that the textual productions presented good quality in the criteria evaluated for both CG and GM. The texts presented few spelling and punctuation errors, sufficient cohesion and coherence, few grammatical errors, variation in the selection of lexical items, avoiding excessive repetition of words, and good organization in terms of structure.

### Quantitative EEG

After processing the EEG data, the sample size was reduced to nine participants from each group. One participant from the GM and the matched control was excluded due to poor quality caused by excessive artifacts in the EEG recordings.

All Box’s M test results for the five frequency bands (delta, theta, alpha, beta 1, beta 2) indicated no significant differences (p > 0.05). Levene’s test also confirmed homogeneity of variances across all bands (p > 0.05). Similarly, Mauchly’s test showed that the assumption of sphericity for repeated measures was met (p > 0.05), supporting the validity of subsequent analyses.

**Fig 3:**
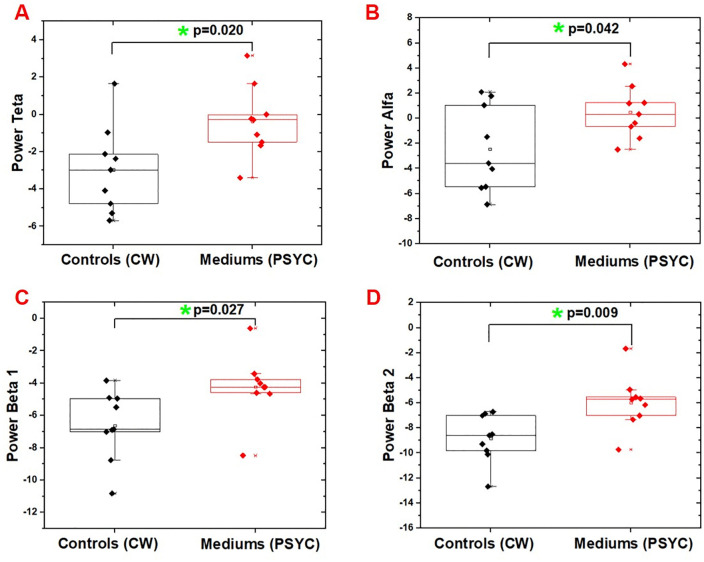
Significant results of the comparison between groups by task. A shows the theta power difference (p = 0.020) between the control group performing the CW task (which simulates psychography) and de mediums performing the PSYC task (written during the mediumistic trance). Similarly, significant differences were also found for other frequency bands: alpha (p = 0.042) in B, beta 1 (p = 0.027) in C, and beta 2 (p = 0.009) in D.

A significant difference between groups was found exclusively in the second writing task, where controls performed the CW task and mediums performed the PSYC task. This result was found in the following frequency ranges: theta, alpha, beta 1, and beta 2 (see Fig 3). For theta, no task-by-group interaction effect was observed [F(1, 16) = 3.737, p = 0.071]. However, Bonferroni-adjusted post hoc comparisons identified a significative difference (p = 0.02) between groups in the second task. There was also no Task *vs.* Group interaction effect [F(1, 16) = 1.419, p = 0.251] for alpha; however, there was a significative difference (p = 0.042) between groups in the post hoc test of the second task. Following this trend, the post hoc test for beta 1 showed a difference (p = 0.027), and there was no Task *vs.* Group interaction effect (F(1, 16) = 2.727, p = 0.118). Similarly, there was no Task *vs.* Group interaction effect in beta 2 [F(1, 16) = 3.411, p = 0.083], though the post hoc test revealed a difference (p = 0.009).

In all frequency ranges with significant results, the mediums exhibited increased power during the PSYC task compared to the CG’s CW task. However, for the WW task, which was common to both groups, there was no difference.

**Fig 4:**
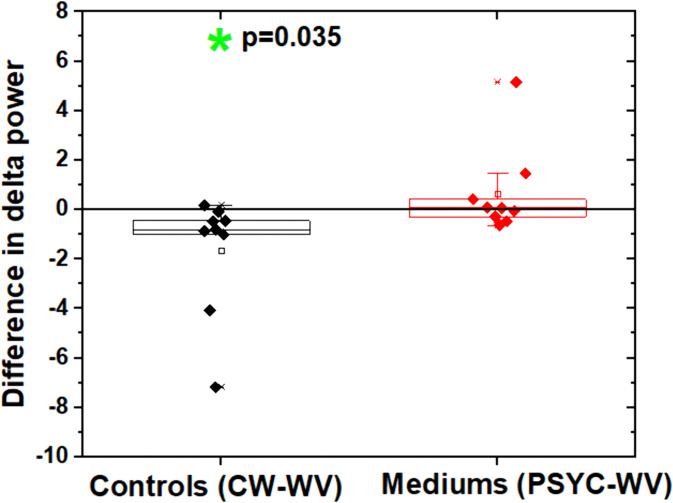
Significant within-group comparison for the CG. The post-hoc analysis revealed a difference in delta power (p = 0.035) between the WW and CW tasks.

Comparisons within each group revealed one difference: between WW and CW tasks in the CG, limited to the delta frequency band (Fig 4). Analysis indicated a task-by-group interaction in the delta range [F(1, 16) = 5.152, p = 0.037]. Bonferroni-adjusted post hoc comparisons confirmed this difference (p = 0.035), showing reduced delta power during CW production (simulated psychography) compared to WW among CG participants. No difference was observed between WW and PSYC tasks in the GM (p = 0.382).

Regarding EEG statistics at the local level (RH and LH), the observed differences mirrored global analysis outcomes, with localized increases in spectral power within the theta, beta 1, and beta 2 bands across both hemispheres during PSYC compared to CW. The only deviation occurred in the alpha band, where a power increase was restricted to RH during PSYC (p = 0.022).

## Discussion

When examining EEG spectral characteristics and linguistic performance during psychography compared to control conditions, group-level analyses revealed increased power in theta, alpha, beta 1, and beta 2 bands during PSYC relative to CW, while WW showed no differences between groups. Textual analysis indicated preserved and even enhanced linguistic complexity during trance. Additionally, delta increased only in the comparison between WW and CW among controls, and local analysis (RH vs. LH) revealed an alpha power increase restricted to RH during PSYC compared to CW. Taken together, these findings do not support the hypothesis that mediumistic trance merely involves increased activity in slow frequencies (delta and theta); rather, they point to a broader cortical reconfiguration and high complexity in language processing.

### qEEG during PSYC

Global qEEG data indicated a distinctivte pattern for PSYC in relation to known associations between brain activity and language. Although no precise EEG spectral signature for verbal language has been established, given that frequency involvement likely varies with task nature [22], studies generally report relative reductions in slower rhythms (delta, theta, alpha) and increased activity in faster rhythms, particularly beta (and, in some contexts, gamma) [24,31,50], which did not occur in PSYC.

Written text production is a complex process requiring synchronization of cognitive, linguistic, and perceptual-motor skills, involving both “central” (cognitive/linguistic) and “peripheral” (motor) processes [6,25,51,52]. The global increase across these frequencies during PSYC, alongside improved text quality, may relate to the distinct functional roles of each band [22,50], whose coexistence and interactions appear necessary for integrating linguistic properties in this type of discursive production [23].

If increased theta may indicate slower brain functioning [27,34–36,53,54], and alpha is considered a reliable marker of reduced cortical arousal [23], these low-frequency bands have also been associated with verbal working memory engagement, essential for phonological elaboration under normal linguistic conditions [22,26,50].

Beta activity (13–30 Hz) tends to increase with task complexity. In this context, beta may function as a marker of cognitive processing, integration, and learning, and has been linked to semantic predictions, syntactic and semantic binding, and motor processing [22,50,55]. Thus, beta-band power modulations capture both linguistic and motor processing, potentially reflecting information flow between these components during writing [55]. In the present study, beta 1 likely represents a transition from slower to faster frequencies in the linguistic domain, while beta 2 has been associated with electrophysiological patterns of linguistic activation [31,55,56], necessary for verbal language emergence and motor execution.

The global and simultaneous power increase across these bands during PSYC may represent a unique activation pattern sustaining expanded resources for complex verbal production such as text writing. Previous EEG studies on mediumship [17–20] also reported increases in these frequencies, though only one described simultaneous increases in theta, alpha, and beta [20], and none involved linguistic tasks. Alterations have also been reported in other trance experiences induced by diverse traditions and practices, including increased theta and alpha [14]; decreased low alpha (8–10 Hz) with increased low beta (13–20 Hz) and high beta (20–30 Hz) [57]; or increased alpha with predominant beta and gamma [58], all compared to ordinary states of consciousness and unrelated to language production.

An additional finding reinforces the characterization of PSYC as a trance experience with a distinct cortical activation pattern compared to other trance states and ordinary consciousness (WW). In intragroup comparisons, the only difference was a delta decrease in CW relative to WW within the CG. For mediums, delta remained stable between PSYC and WW, despite PSYC involving cognitive effort to write as a distinct (deceased) personality and producing higher text quality, as confirmed by statistical differences in evaluations. CW likely demands greater cognitive effort, requiring memory retrieval and articulation of information about another person and adaptation of textual style. Considering delta is associated with inhibitory processes in cognitive and linguistic performance [28,53,54], its reduction in CW compared to WW among controls aligns with literature.

### Local analysis (RH *vs*. LH)

Local hemispheric data showed PSYC with simultaneous increases in theta, beta 1, and beta 2 across both hemispheres compared to CW, with alpha increasing only in RH. Functional lateralization appears necessary for full linguistic potential [31]. Linguistic tasks emphasizing phonological processing exhibit higher theta and alpha in RH compared to LH, reflecting active inhibition of non-linguistic regions [27,31,34,59]. Linguistic performance is also associated with beta increases and greater LH lateralization [27,31], though writing tasks additionally show beta increases in RH linked to motor action [55]. Thus, PSYC may represent a peculiar hemispheric pattern, as no clear dominance emerged. Alpha increase in RH, if linked to reduced cortical activity, could suggest relative LH prevalence during PSYC, aligning with common findings.

### Limitations and future directions

The sample size was limited and based on convenience, which restricts generalizability and reduces statistical power for regional analyses (e.g., lobar-level comparisons) and increases susceptibility to individual variability. Therefore, the present findings should be interpreted as exploratory and preliminary, serving primarily to generate hypotheses for future studies with larger samples. Although pre-resting-state EEG data was available, it was not analyzed in this study. This prevented between-group baseline comparisons and the assessment of residual effects in the current work, which remains a goal for future studies. Future studies should incorporate pre- and post-task resting-state recordings to evaluate lingering effects of task engagement. Additionally, subsequent research should to include measures to capture immediate state transitions (pre- and pos-trance) and refine understanding of neural dynamics underlying mediumistic trance.

One of the mediums presents unique characteristics regarding “loss of consciousness,” given the maximum score assigned to this experience (Supplementary Material, S5 in S2 File). Phenomenologically, this may indicate alterations in attention and sense of control, accompanied by partial amnesia [6,12,14]. Studies [60–62] report significant increases in slow rhythms, alongside simultaneous increases in fast rhythms during states of reduced consciousness. Despite phenomenological differences (mediumistic trance versus coma), co-modulation across bands suggests that trance depth may differentially modulate oscillatory patterns. This is noteworthy given the high complexity of writing in both linguistic and motor domains. Writing is a complex act that requires the synchronization of linguistic, motor, and spatial skills supported by widespread brain activity [51,59]. Given these specificities, characterizing trance in relation to potential reductions in the level of consciousness emerges as a relevant criterion for future investigations.

## Conclusion

This study provides novel evidence that mediumistic trance writing, an underexplored cultural and cognitive phenomenon, can sustain complex linguistic production while engaging distinctive neural oscillatory dynamics. Unlike the canonical EEG pattern for language tasks, which typically shows reduced slow rhythms and increased beta activity, psychography revealed simultaneous enhancement of theta, alpha, and beta bands, suggesting a unique cortical reconfiguration rather than a simple shift in frequency dominance. These findings challenge conventional assumptions about the limits of language processing in non-ordinary states of consciousness and open new avenues for neurophenomenological research. Future studies integrating connectivity analyses, larger samples, and pre/post-trance baselines will be essential to elucidate how altered states interact with high-level cognitive functions such as writing.

## Supporting information

S1 File Supplementary materials. This file includes all supporting materials referenced in the manuscript: S1 – the NEUPSILIN, SCID-5, PANSS, and DES assessments; S2 – the pre-collection questionnaire; S3 – the questionnaire evaluating mediumistic trance quality; S4 – the text evaluation model and scoring criteria.
(DOCX)

S2 File S5 – the post-collection trance evaluation data and figure. (TIF)
