# Supplementary material for: Brain activity and the production of written text during mediumistic trance: A controlled EEG study
Source: PLoS One. 2026 Feb 24;21(2):e0343216. doi: 10.1371/journal.pone.0343216 (PMC12931803; doi:10.1371/journal.pone.0343216)
Supplement: S1 File — This file includes all supporting materials referenced in the manuscript: S1 – the NEUPSILIN, SCID-5, PANSS, and DES assessments; S2 – the pre-collection questionnaire; S3 – the questionnaire evaluating mediumistic trance quality; S4 – the text evaluation model and scoring criteria. (DOCX) [file pone.0343216.s001.docx]

**SUPPLEMENTARY MATERIAL**

**Brain activity and the production of written text during mediumistic trance: a controlled EEG study**

***Kleber Monteiro Pinto ^1#a, 2*^, Thaise G. L. de O. Toutain ^1, 3, 4^, Hugo Saba ^5, 6^, Marco Aurélio Vinhosa Bastos Jr. ^7^, Raphael Silva do Rosário ^1, 8^, Jéssica Plácido ^9^, Naíma Loureiro ^1, 10^, Valéria C. Fernandes ^11, 12^, José Garcia Vivas Miranda ^1, 8^***

*^1^ Laboratory of Biosystems, Federal University of Bahia, Brazil;*

*^2^ Department of Education, Campus I, State University of Bahia, Brazil;*

*^3^ School of Health and Natural Sciences, Catholic University of Salvador, Bahia, Brazil;*

*^4^ Institute of Mathematical and Computer Sciences, University of São Paulo, São Paulo, Brazil;*

*^5^ Department of Exact and Earth Sciences, State University of Bahia, Brazil;*

*^6^ Senai Cimatec University, Salvador, Bahia, Brazil;*

*^7^ School of Medicine – Federal University of Mato Grosso do Sul, Brazil;*

*^8^ Department of Earth and Environmental Physics, Federal University of Bahia,*

*Salvador, Brazil*

*^9^ Bahiana School of Medicine and Public Health, Bahia, Brazil;*

*^10^ Health Sciences Institute, Federal University of Bahia, Salvador, Bahia, Brazil;*

*^11^ Electrochemistry, Energy and Materials Research Group (GPEEM), Federal University of Bahia, Institute of Chemistry, Department of Physical Chemistry. Salvador, Bahia, Brazil;*

*^12^ Postgraduate Program in Energy and Environment (PGEnAm), Federal University of Bahia, Brazil.*

#a Current Address: *Department of Education, Campus I, State University of Bahia.*

* Corresponding author

E-mail: kpinto@uneb.br

Here we present the questionnaires used in this study and some of the results obtained.

**S1.  NEUPSILIN, SCID-5, PANSS and DES**

NEUPSILIN: Brief Neuropsychological Assessment. According to the scores compared with normative data by age group and education, the results for the group of mediums (GM) did not indicate any cognitive deficit or below-average performance for a healthy neuropsychological profile in the evaluation of the 8 preserved functions: temporospatial orientation, attention, perception, memory (Toutain, 2024), arithmetic skills, language, praxias, and executive function (problem-solving and verbal fluency).

SCID-5-CV. For the evaluation of each diagnostic criterion for the main mental disorders according to DSM-5, symptoms were coded as "NO"/"YES" during the interview. After assessing all relevant modules, the GM did not meet the criteria for any of the mental disorders included in the interview. However, some of the GM's results concerning schizophrenia, which is part of the psychotic disorders module, showed variations in the assessment of positive symptoms (delusions, hallucinations, disorganized speech, catatonic behavior) (Toutain, 2024).

In this context, the observed differences correspond to hallucinatory features assessed in Module B. The majority of the mediums reported affirmative responses to items B14 through B19, which characterize episodes of sensory experiences occurring in the absence of appropriate stimulation of the respective sensory organs and that are generally not shared by people in the surrounding environment (Toutain, 2024). The answers of most participants reported hallucinatory experiences, particularly visual (10/10) and auditory hallucinations (9/10). Perceptual hallucinations were less frequent (6/10), while sensations of electricity on the skin and unpleasant smells were reported by half of the participants (5/10) (Toutain, 2024).

None of the GM participants presented negative symptoms, such as affective flattening, avolition, or anhedonia. Despite showing hallucinatory features, all mediums responded “No” to the Module B items designed to screen for delusional symptoms (B1–B13) (Toutain, 2024).

PANSS. On item P3, which assesses hallucinatory perceptions, the GM showed the only notable variation, with a mean (SD) of 4 (1) (Toutain, 2024). Ratings were based on a 7-point scale ranging from absent (1) to extreme (7) (Leucht et al., 2005).

DES: Dissociative Experiences Scale. The use of this instrument screens for the subcomponents of dissociation across four factors: absorption, depersonalization, moderate memory disturbances, and amnesia. A DES score of 30 is considered the cutoff point above which it's possible to screen for patients with a dissociative disorder. Two individuals of the GM showed non-pathological dissociation, and none from the control group (CG) (Toutain, 2024).

**S2. Pre-collection questionnaire**

Read the affirmative sentences below and mark the answers "YES" or "NO". Answer honestly.

| I slept at least 8 hours the night before. | ^YES^ | ^NO^ |
| --- | --- | --- |
| I washed my hair with coconut soap before coming to pick it up. | ^YES^ | ^NO^ |
| I drank coffee or another stimulant beverage today. | ^YES^ | ^NO^ |
| I feel ready to participate in the collection today. | ^YES^ | ^NO^ |
| I feel comfortable participating in the collection today. | ^YES^ | ^NO^ |
| I signed the Informed Consent Form. | ^YES^ | ^NO^ |

**S3. Questionnaire evaluating the quality of the mediumistic trance experience**

| **TEXT nº  (Evaluator nº )** | | | | |
| --- | --- | --- | --- | --- |
|  | 11 | 22 | 33 | 44 |
| I - Orthographic accuracy: spelling, accentuation, and punctuation |  |  |  |  |
| II - Lexical item selection: variation in the use of different words and figures of speech |  |  |  |  |
| III - Grammatical accuracy: verb conjugation, government, and verbal and nominal agreement |  |  |  |  |
| IV - Text organization: division into sentences and paragraphs (if any) and structure, including introduction, development, and conclusion |  |  |  |  |
| V - Textual cohesion: referential, recurrent, and sequential cohesion elements |  |  |  |  |
| VI - Textual coherence: continuity of meaning, ensuring consistency between concept configuration and expressed relationships |  |  |  |  |

**S4. Text Evaluation Model**

**Criteria used to assess written content:**

- Orthographic accuracy – spelling, accentuation, and punctuation;
- Lexical item selection – variation in the use of different words and figures of speech;
- Grammatical accuracy – verb conjugation, government, and verbal and nominal agreement;
- Text organization – division into sentences and paragraphs, and structure including introduction, development, and conclusion;
- Textual cohesion – elements of referential, recurrent, and sequential cohesion;
- Textual coherence – continuity of meaning, ensuring consistency between the configuration of concepts and their expressed relationships.

Scores ranged from 1 to 4 for each criterion, as follows: (1) Insufficient, (2) Fair, (3) Good, and (4) Very good.

**Score Assignment**

- Orthographic accuracy – spelling, accentuation, and punctuation: (1) Insufficient: 16 or more errors; (2) Fair: 8 to 15 errors; (3) Good: 3 to 7 errors; (4) Very good: at most 2 errors.
- Lexical item selection – variation in the use of different words and figures of speech:
  (1) Insufficient: more than 50% repetition of meaningful words and textual connectors;
  (2) Fair: 30% to 50% repetition of meaningful words and textual connectors;
  (3) Good: 20% to 30% repetition of meaningful words and textual connectors;
  (4) Very good: less than 20% repetition of meaningful words and textual connectors, with use of figures of speech.
- Grammatical accuracy – verb conjugation, government, and verbal and nominal agreement: (1) Insufficient: more than 10 errors; (2) Fair: 6 to 10 errors; (3) Good: 1 to 5 errors; (4) Very good: no errors.
- Text organization – division into sentences and paragraphs, and structure including introduction, development, and conclusion: (1) Insufficient: no division or structure; (2) Fair: no paragraph division and missing one structural element; (3) Good: paragraph division but missing one structural element; (4) Very good: paragraph division and all structural elements present.
- Textual cohesion – referential, recurrent, and sequential cohesion elements: (1) Insufficient: cohesion elements in less than 50% of sentences and paragraphs; (2) Fair: cohesion elements in 50% to 80% of sentences and paragraphs; (3) Good: cohesion elements in more than 80% of sentences and paragraphs; (4) Very good: cohesion elements in all sentences and paragraphs.
- Textual coherence – continuity of meaning, ensuring consistency between concept configuration and expressed relationships: (1) Insufficient: continuity and consistency in less than 50% of sentences and paragraphs; (2) Fair: continuity and consistency in 50% to 80% of sentences and paragraphs; (3) Good: continuity and consistency in at least 80% of sentences and paragraphs; (4) Very good: continuity and consistency throughout all sentences, paragraphs, and the text as a whole.

**Sample excerpts of texts**

The texts below present representative samples from each task, obtained from one medium and the corresponding control; all proper names have been removed to preserve anonymity. All texts were written in Portuguese and are presented here translated into English.

1 - Group of mediums

1.1 - Waking Writing (WW)

My love,

Daddy is taking part today in an interesting research study on mediumship. I’m missing you terribly, of course, but I’ll be back very soon. When you grow a little older, you’ll be able to come along with me on these trips, and then it will be amazing.

Actually, that was all I wanted to tell you, but I need to keep writing until the researcher tells me to stop.

I hope your Sunday is beautiful and very happy.

With all the love in the world, Daddy.

P.S. The exercise isn’t over yet. I’ll take this opportunity to say that I’ve gotten to know the city of Salvador a bit more, and I still plan to take some other trips. If I find them, I’ll bring you *cocada* and *cupuaçu*. I haven’t tried it yet, but it’s delicious—tangy and slightly sour. Where we live, it’s hard to find.

That’s all.

1.2 - Psychography (PSYC)

It is necessary to reduce the distances between the different planes of the spirit’s existence, and to this end, it is both necessary and desirable to seriously re-found the paradigm of a materialistic science that is incapable of understanding the expanded horizons of the mind and of thought.

To think about the incarnate subject is to contemplate, simultaneously, biology and psychism, making indispensable the intermediate instance that spiritual traditions call the *perispirit*, but which finds analogues in various “psy” schools under the guise of foundational or constitutive psychic records—specifically referred to as proto-phantasies, remnants, or verbal traces. This reflects the interdependence of phylogenetic fields inherited from the species, manifesting themselves in the ontogenesis of individuals.

What is not proven is the existence of the spirit as a mere abstraction; rather, what is demonstrated is that there exists within the human being—beyond any reduction to the brain—an intricate network of consciousness, sufficiently sophisticated to produce realities. From this standpoint arise our spiritual readings of contemporary psychic research.

We hope to walk alongside you on this journey.

CW

2 - Control group

2.1 - Waking Writing (WW)

My dear grandfather,

I wish I could hear from you. I often wonder how things are there, on the other side of life—as I have learned to call it.

More than once since you passed away, I have dreamed of you, and at times, I had the impression that you had already adapted to your new reality and were even working there, in the spiritual realm. I hope that this is true.

Here, we are all doing well. We miss you, as anyone who had a father or grandfather like you would.

With love,

Your grandson.

 2.2 - Creative Writing

My friends,

The one who speaks to you here is the good old CX. I know that at this very moment you are engaged in the noble task of scientific research, about which I myself know very little or perhaps nothing at all. You see, I have always served as the one who would be tested, so now, as I observe you, I understand little of all that you do. But I do know that you are in the service of good, working toward the construction of a world in which the lights of reason do not overshadow, but rather join with, the lights of faith. For this reason, I greet you and congratulate you on the good seed you are planting.

With affection,

From your dear

CX

**S5. Post-collection questionnaire**

Administered to each participating medium to evaluate their perception of the mediumistic trance experience, according to the following criteria:  Sense of comfort and confidence; Spirituality-related sensations; Involuntary movements; Loss of consciousness; and Trance quality compared to their usual experience. Participants were evaluated on a scale of 0 to 5, with 0 meaning non-existent or awful and 5 meaning complete, consistent, or excellent.

**S5 Fig. Trance evaluation. Each bar shows the individual score of participants (1–9) for five dimensions of the mediumistic trance experience. Responses were recorded on a 0–5 scale and bar colors indicate the evaluated criteria as shown in the legend.**

Composition of each medium's scores by criterion. Scores for the sensation of comfort were high; one medium scored 4, and all others scored the maximum (5). Similarly, trance quality scores were also high: five mediums scored the maximum (5), three others scored 4, and only one scored 3. For the sensation of spirit presence criterion, everyone scored the maximum (5). However, in the involuntary movements criterion, there was a variation in participants' experiences: three scored the maximum (5), one scored 3, one scored 2, and four mediums scored 0, indicating no involuntary movements. For loss of consciousness (represented in yellow), there was only one high score at the maximum value (5), with only two other participants scoring 1, while the rest scored 0.

**REFERENCES**

Toutain TGLO. Características funcionais da conectividade cerebral em estados alterados de consciência [Functional characteristics of brain connectivity in altered states of consciousness] [PhD Thesis]. Salvador (BA): Federal University of Bahia. Available from <https://repositorio.ufba.br/handle/ri/39486>

Leucht S, Kane JM, Kissling W, Hamann J, Etschel E, Engel RR. What does the PANSS mean? Schizophrenia research. Schizophrenia Research. 2005; 79(2-3): 231-238. doi: [10.1016/j.schres.2005.04.008](https://doi.org/10.1016/j.schres.2005.04.008)
